# High-throughput screening identifies established drugs as SARS-CoV-2 PLpro inhibitors

**DOI:** 10.1007/s13238-021-00836-9

**Published:** 2021-04-17

**Authors:** Yao Zhao, Xiaoyu Du, Yinkai Duan, Xiaoyan Pan, Yifang Sun, Tian You, Lin Han, Zhenming Jin, Weijuan Shang, Jing Yu, Hangtian Guo, Qianying Liu, Yan Wu, Chao Peng, Jun Wang, Chenghao Zhu, Xiuna Yang, Kailin Yang, Ying Lei, Luke W. Guddat, Wenqing Xu, Gengfu Xiao, Lei Sun, Leike Zhang, Zihe Rao, Haitao Yang

**Affiliations:** 1 Shanghai Institute for Advanced Immunochemical Studies and School of Life Science and Technology, ShanghaiTech University, Shanghai, 201210 China; 2 Laboratory of Structural Biology, School of Life Sciences and School of Medicine, Tsinghua University, Beijing, 100091 China; 3 State Key Laboratory of Virology, Wuhan Institute of Virology, Center for Biosafety Mega-Science, Chinese Academy of Sciences, Wuhan, 430071 China; 4 The Fifth People’s Hospital of Shanghai, Fudan University and Shanghai Key Laboratory of Medical Epigenetics, Institutes of Biomedical Sciences, Fudan University, Shanghai, 200032 China; 5 Zhangjiang Lab, National Facility for Protein Science in Shanghai, Shanghai Advanced Research Institute, Chinese Academy of Science, Shanghai, 201210 China; 6 Taussig Cancer Center, Cleveland Clinic, Cleveland, OH 44195 USA; 7 School of Chemistry and Molecular Biosciences, The University of Queensland, Brisbane, Queensland 4072 Australia

**Keywords:** SARS-CoV-2, papain-like protease, YM155, interferon stimulating gene product 15, drug repurposing

## Abstract

A new coronavirus (SARS-CoV-2) has been identified as the etiologic agent for the COVID-19 outbreak. Currently, effective treatment options remain very limited for this disease; therefore, there is an urgent need to identify new anti-COVID-19 agents. In this study, we screened over 6,000 compounds that included approved drugs, drug candidates in clinical trials, and pharmacologically active compounds to identify leads that target the SARS-CoV-2 papain-like protease (PLpro). Together with main protease (M^pro^), PLpro is responsible for processing the viral replicase polyprotein into functional units. Therefore, it is an attractive target for antiviral drug development. Here we discovered four compounds, YM155, cryptotanshinone, tanshinone I and GRL0617 that inhibit SARS-CoV-2 PLpro with IC_50_ values ranging from 1.39 to 5.63 μmol/L. These compounds also exhibit strong antiviral activities in cell-based assays. YM155, an anticancer drug candidate in clinical trials, has the most potent antiviral activity with an EC_50_ value of 170 nmol/L. In addition, we have determined the crystal structures of this enzyme and its complex with YM155, revealing a unique binding mode. YM155 simultaneously targets three “hot” spots on PLpro, including the substrate-binding pocket, the interferon stimulating gene product 15 (ISG15) binding site and zinc finger motif. Our results demonstrate the efficacy of this screening and repurposing strategy, which has led to the discovery of new drug leads with clinical potential for COVID-19 treatments.

**Supplementary Information:**

The online version contains supplementary material available at 10.1007/s13238-021-00836-9.

## Introduction

Coronaviruses (CoVs) are a large family of positive-stranded RNA viruses that, in recent times, have caused severe acute respiratory syndrome (SARS) and Middle East respiratory syndrome (MERS) (de Wit et al., 2016). In December 2019, a newly identified coronavirus, severe acute respiratory syndrome coronavirus 2 (SARS-CoV-2), was identified as the etiological agent responsible for the outbreak of COVID-19 (World Health Organization, 2020). Symptoms associated with COVID-19 include fever, cough, fatigue, nausea and shortness of breath, with the severity ranging from mild to fatal (Zhou et al., 2020a). Considering the current global pandemic, there is an urgent need to develop effective antiviral agents to treat COVID-19 (Sanders et al., 2020).

SARS-CoV-2 belongs to the *Betacoronavirus* genus, which also contains SARS-CoV and MERS-CoV (Zhou et al., 2020b). The RNA genome of SARS-CoV-2 is comprised of ~30,000 nucleotides, with the replicase gene occupying two-thirds of the 5′ end of the viral genome. The replicase gene encodes two long overlapping polyproteins, pp1a and pp1ab. In the SARS-CoV-2 life cycle, processing of pp1a and pp1ab into 16 non-structural proteins (Nsps) is a critical step required for RNA transcription and genome replication. Proteolytic processing is achieved by two cysteine proteases, the papain-like protease (PLpro) and the main protease (M^pro^, also known as 3CLpro) (Jin et al., 2020a; Jin et al., 2020b). PLpro is responsible for releasing Nsp1-Nsp3 from the N-terminus of this polyprotein by recognizing the consensus sequence, LXGG (Barretto et al., 2005a). Additionally, PLpro catalyzes deubiquitination and removal of interferon stimulating gene product 15 (ISG15) from host proteins, thereby interfering with the host innate immune response (Bailey-Elkin et al., 2014; Daczkowski et al., 2017; Shin et al., 2020). Given its essential role in viral replication, PLpro is considered an attractive target for antiviral drug development (Sanders et al., 2020).

Repurposing is an effective strategy in drug discovery, an approach that has the ability to accelerate deployment of drugs to the marketplace. For rapidly spreading infectious diseases such as COVID-19, clinical trials can be carried out promptly to identify novel leads to contain a pandemic. In this study, we designed an approach using a SARS-CoV-2 PLpro inhibition activity as the primary screening tool for the discovery of the small molecule inhibitors, with a secondary cell-based assay for evaluation of their antiviral activity. High-throughput screening of over 6,000 compounds enabled us to identify four compounds that strongly inhibit SARS-CoV-2 PLpro. These are YM155, cryptotanshinone, tanshinone I and GRL0617. In the antiviral assays, all four compounds showed inhibition of SARS-CoV-2 replication in Vero E6 cells, suggesting their potential for clinical development to treat COVID-19. Among these compounds, YM155, an anticancer drug candidate in clinical trials, has the most potent antiviral activity. In addition, we have determined the crystal structures of the SARS-CoV-2 PLpro^C111S^ and its complex with YM155, revealing a unique binding mode where YM155 simultaneously targets three “hot” spots on PLpro.

## Results

### High-throughput drug repurposing screening for SARS-CoV-2 PLpro

Recombinant SARS-CoV-2 PLpro expressed in *Escherichia coli* was purified to homogeneity (Fig. S1A and S1B). In order to characterize its enzymatic activity and subsequently carry out high-throughput screening of inhibitors, we utilized a fluorescence-based enzymatic assay. A fluorescently labeled substrate (Ratia et al., 2008), Arg-Leu-Arg-Gly-Gly-AMC (AMC: aminomethyl-coumarin), derived from the natural substrate for PLpro and other deubiquitinating enzymes, was synthesized for use in the kinetic assays. The catalytic efficiency (*k*_*cat*_/*K*_*m*_) of this substrate for SARS-CoV-2 PLpro was measured to be 1,840 s^−1^·mol/L^−1^ (Fig. S1C).

**Figure 1:**
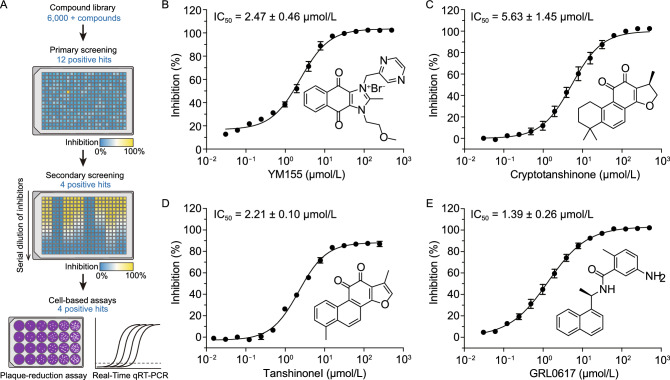
**Schematic diagram of the high-throughput screening process to discover COVID-19 drug leads**. (A) Schematic of the high-throughput screening and hit validation process. (B–E) Drug leads inhibiting the SARS-CoV-2 PLpro. The hydrolytic activity of SARS-CoV-2 PLpro was measured in the presence of varying concentrations of the drug candidates. Dose-response curves for IC_50_ values of YM155 (B), cryptotanshinone (C), tanshinone I (D) and GRL0617 (E) were determined by nonlinear regression. Data were shown as mean ± s.e.m., *n* = 3 biological replicates

Next, over 6,000 compounds from libraries consisting of approved drugs, drug candidates in clinical trials and pharmacologically active compounds were screened against SARS-CoV-2 PLpro to identify new inhibitors (Fig. 1A). Their half-maximal inhibitory concentrations (IC_50_) values were then determined (Fig. 1B–E). Primary hits included 12 compounds that showed >60% inhibition against PLpro when assayed at a concentration of 50 µmol/L. After examining the structure of each hit compound, we removed compounds containing reactive functional groups or those with known cytotoxicity. After exclusions, four compounds (YM155, cryptotanshinone, tanshinone I and GRL0617) were selected (Fig. 1B–E) for further assessment. A detergent-based assay (Feng and Shoichet, 2006) was used as a control to assess if any of these are aggregate-based inhibitors (Fig. S2). YM155, an antineoplastic drug in clinical trials, inhibited PLpro with an IC_50_ value of 2.47 μmol/L. Cryptotanshinone and tanshinone I, the active ingredients from Chinese herbal medicine, *Salvia miltiorrhiza,* inhibited PLpro with IC_50_ values of 5.63 and 2.21 μmol/L, respectively. GRL0617, a compound previously reported to inhibit SARS-CoV PLpro (Ratia et al., 2008), also inhibits SARS-CoV-2 PLpro with an IC_50_ value of 1.39 μmol/L, in agreement with a recent study (Shin et al., 2020).

### Antiviral activity and cytotoxicity assays

**Figure 2:**
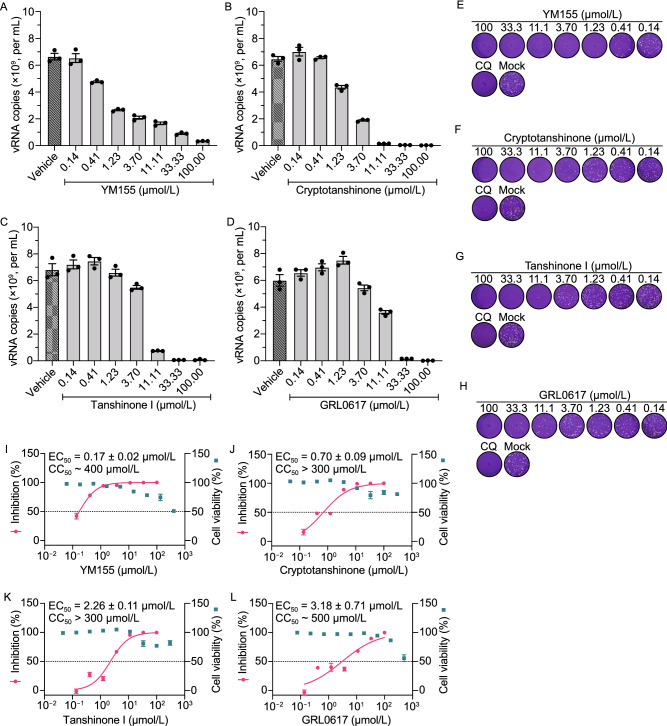
**Antiviral activities of YM155, cryptotanshinone, tanshinone I and GRL0617 against SARS-CoV-2**. (A–D) The quantification of absolute viral RNA copies (per mL) in the supernatant at 24 h post infection determined by qRT-PCR analysis. Data are shown as mean ± s.e.m., *n* = 3 biological replicates. (E–H) Images for the plaque-reduction assay. As the concentration of indicated drugs increases, there is a substantial reduction in the numbers of the plaques by comparison with negative control (Mock). Chloroquine (CQ) was used as a positive control. Results are shown as representative of three biological replicates. (I–L) Dose-response curves of the indicated antivirals in the plaque-reduction assay (EC_50_) and cytotoxicity (CC_50_) to Vero E6 cells measured by CCK-8 assays. The left and right Y-axis of the graphs represent mean inhibition (%) of virus yields and cytotoxicity of the drugs, respectively. Data are shown as mean ± s.e.m., *n* = 3 biological replicates

To further substantiate these compounds as antiviral drug leads, we evaluated whether these four compounds could prevent SARS-CoV-2 replication at the cellular level. First, quantitative real-time RT-PCR (qRT-PCR) for viral RNA copy numbers in the cellular supernatant demonstrated that all these four compounds exhibited strong antiviral effects (Fig. 2A–D). Further, a plaque-reduction assay (Fig. 2E–H) was performed to accurately determine the half-maximal effective concentration (EC_50_) values. The four compounds inhibited SARS-CoV-2 with EC_50_ values ranging from 0.17 to 3.18 μmol/L (Fig. 2I–L). Thus, all four compounds penetrate the cellular membrane to access their targets.

**Figure 3:**
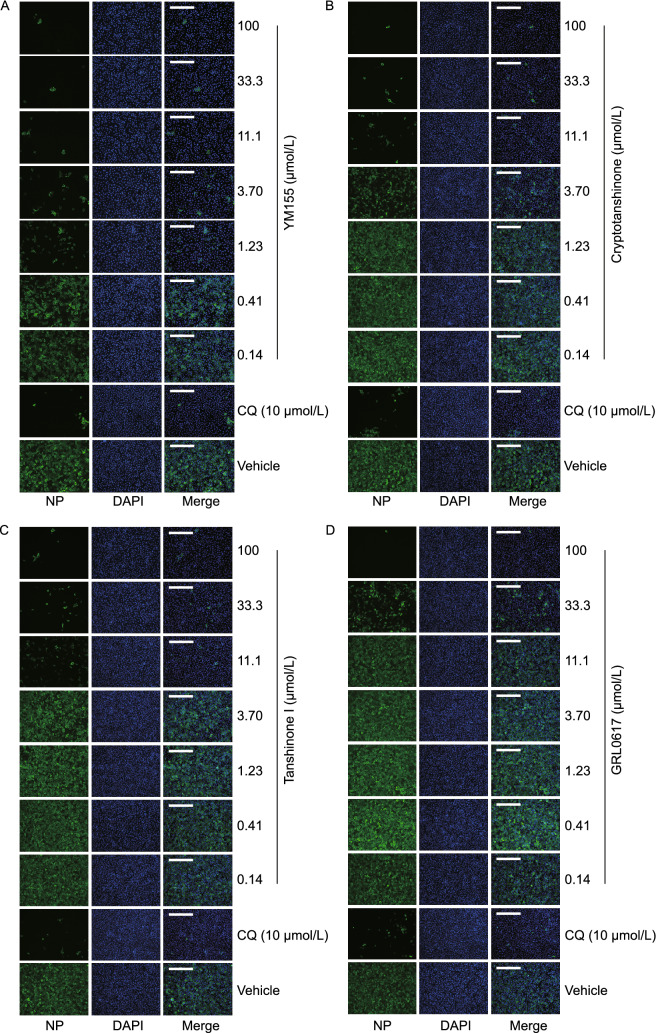
**Immunofluorescence microscopy of SARS-CoV-2 infection upon treatment of the lead compounds**. (A–D) At 24 h post infection, the infected cells were fixed and intracellular NP levels were monitored by anti-NP rabbit sera (primary antibody) and Alexa 488-labeled secondary antibody. The nuclei were stained with DAPI. Scale bars, 400 μm. Chloroquine (CQ, 10 μmol/L) was used as a positive control. The results are representative of three biological replicates

The antiviral effect of the compounds was further evidenced by immunofluorescence staining of infected cells using SARS-CoV-2 nucleocapsid protein (NP) anti-sera. We found that the NP levels decreased with an increase in the concentration of all the compounds (Fig. 3). Notably, at the drug concentration of 11.1 μmol/L, cryptotanshinone and tanshinone I showed comparable NP levels with the control group (10 μmol/L chloroquine, CQ); YM155 achieves a similar inhibition level, but at a much lower concentration (1.23 μmol/L). The cytotoxicity of these compounds was determined in Vero E6 cells using the CCK-8 assay (Fig. 2I–L). Their 50% cytotoxicity concentration (CC_50_) values are all above 300 μmol/L.

In a previous study, chloroquine and remdesivir were shown to have strong inhibition towards SARS-CoV-2 in cell-based assays (Zhou et al., 2020b). However, a large-scale study reports that chloroquine or hydroxychloroquine (used alone or with a macrolide) given to patients hospitalized with COVID-19 increases the risk of death compared with those who were not given this drug (Mehra et al., 2020). Remdesivir, an inhibitor of the viral RNA-dependent RNA polymerase (RdRp), was identified as a promising therapeutic candidate for COVID-19 (Beigel et al., 2020; Goldman et al., 2020), but the level of effectiveness of remdesivir remains unclear (Wang et al., 2020). Food and Drug Administration (FDA) in U.S.A has approved remdesivir to treat COVID-19, for use in adults and pediatric patients (12 years of age and older and weighing at least 40 kg) requiring hospitalization (Hinton, 2020). However, a large study led by the World Health Organization suggests that the remdesivir did not help hospitalized COVID-19 patients (Keaten and Marchione, 2020). In this study, YM155 (EC_50_ = 170 nmol/L) displays more potent inhibition than chloroquine (EC_50_ = 1.13 μmol/L) or remdesivir (EC_50_ = 0.77 μmol/L) (Zhou et al., 2020b), while cryptotanshinone, with an EC_50_ of 0.70 μmol/L, has a similar antiviral activity to that of remdesivir.

### Crystal structure of SARS-CoV-2 PLpro^C111S^

**Figure 4:**
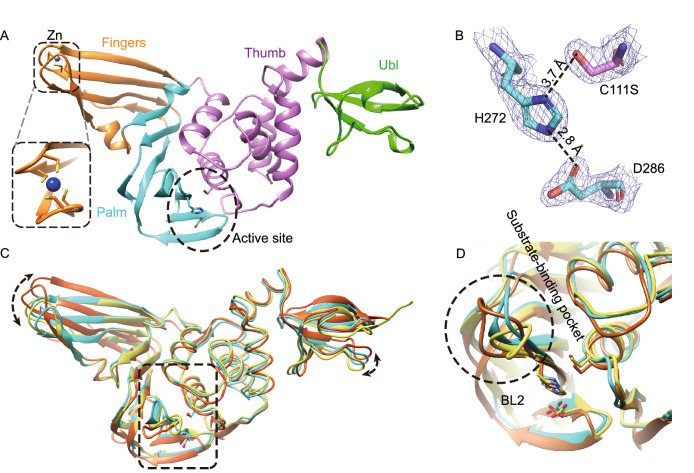
**Crystal structure of SARS-CoV-2 PLpro**^**C111S**^. (A) Cartoon representation of SARS-CoV-2 PLpro^C111S^ polypeptide structure with each domain colour-coded. The catalytic triad is shown as sticks. The zinc ion (shown as a blue sphere) is coordinated by four cysteine residues (shown as sticks). The zinc-ion binding area is shown in the left-bottom panel for clarity. (B) Electron density of the catalytic triad. A *2Fo-Fc* map is contoured at 1.8σ (blue). (C) Comparison of SARS-CoV-2 PLpro (cyan) with the SARS-CoV PLpro (yellow, PDB ID 2FE8) and MERS-CoV PLpro (orange, PDB ID 4RNA). The structural differences at the Ubl domain and the zinc-binding region are apparent (marked by dashed arrows). (D) Magnified view of the substrate-binding pockets. The conserved catalytic triad residues are shown by ball-and-stick representations. The flexible BL2 region is marked by a dashed circle

To better understand the molecular basis of inhibition, we determined the structure of SARS-CoV-2 PLpro. Of all the trials to crystallize the wild type and mutant SARS-CoV-2 PLpro proteins, the SARS-CoV-2 PLpro^C111S^ (4–315) construct gave the best crystals, allowing a 1.9 Å resolution structure to be determined. All of the residues are visible in the electron density maps. The PLpro monomer consists of an independent N-terminal ubiquitin-like (Ubl) domain (residue 1–61) and a catalytic region with a right-handed thumb–palm–fingers architecture (Fig. 4A). Although gel filtration analyses indicated that PLpro is predominately monomeric in solution, PLpro^C111S^ crystallized in the space group *P*6_5_22 with two polypeptides in the asymmetric unit (Fig. S4A). These two molecules (A and B) superimpose with a root mean square deviation (r.m.s.d.) of 0.65 Å for all Cα atoms (Fig. S4B). Thus, there is no overall difference between the two polypeptide structures. Locally, the angle between the N-terminal Ubl domain and the catalytic region is different in the two structures, suggesting the flexibility of the connection between two domains (Fig. S4B).

The overall structure of PLpro of SARS-CoV2 is similar to other CoV PLpro structures (Fig. 4C). The r.m.s.d. between the equivalent Cα atoms of SARS-CoV-2 PLpro and SARS-CoV PLpro (PDB ID 2FE8) is 1.1 Å and there is 82% sequence identity (Ratia et al., 2006). The SARS-CoV-2 PLpro and MERS-CoV PLpro (PDB ID 4RNA) structures are also similar (r.m.s.d. 2.1 Å), even though they share much lower sequence identity (29%) (Lei and Hilgenfeld, 2016) (Fig. S3). Four cysteine residues at the finger-tips coordinate a zinc ion with tetrahedral geometry to constitute a zinc finger motif (Fig. 4A), which is essential for structural integrity and protease activity (Herold et al., 1999; Barretto et al., 2005b).

Thumb and palm domains are the most structurally conserved regions among PLpros from different CoVs (Fig. 4C). The substrate-binding pocket of SARS-CoV-2 PLpro is located at the interface of the thumb and palm domains. It contains a conserved classic catalytic triad (C111–H272–D286), which adopts a consistent conformation among different PLpro proteins (Fig. 4B). Mutating C111 to serine did not induce a conformational change of the active site (Figs. 4D and S5). Access to the active site is regulated by a flexible blocking loop 2 (BL2), which is also involved in substrate binding. Large structural variations in this loop are observed among different PLpro proteins (Fig. 4D).

### Crystal structure of SARS-CoV-2 PLpro^C111S^ -YM155

**Figure 5:**
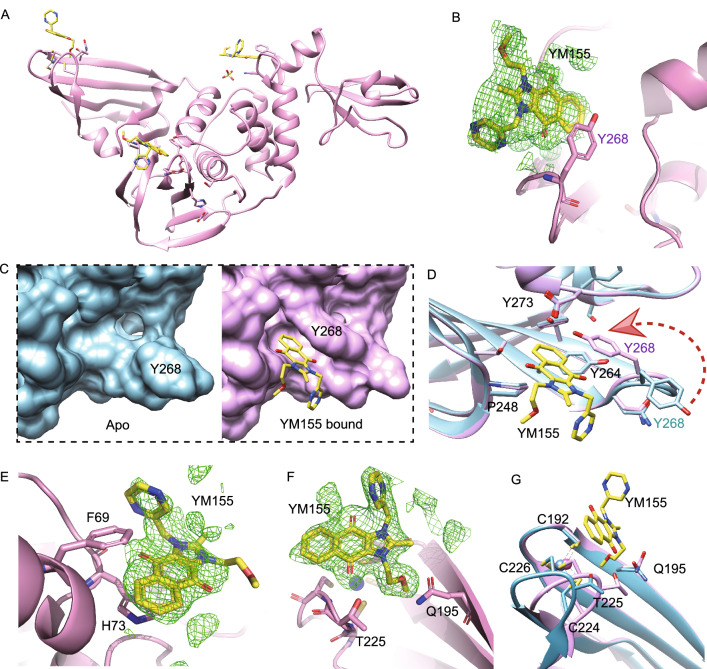
**Crystal structure of SARS-CoV-2 PLpro**^**C111S**^**-YM155 complex**. (A) The monomeric structure of SARS-CoV-2 PLpro^C111S^-YM155 (molecule B), showing three YM155 molecules bound (shown as yellow sticks). (B) Electron density of YM155 molecule located at the substrate-binding pocket. The omit *Fo-Fc* map is contoured at 1.5σ (green). (C) Comparison of the substrate-binding pocket of apo (light blue) and YM155 bound (pink). (D) Superposition of the substrate-binding pocket of apo (light blue) and YM155 bound (pink). (E) Electron density for YM155 located at the thumb domain. The omit *Fo-Fc* map contoured at 1.5σ (green). (F) Electron density for YM155 located at the zinc-finger motif. The omit *Fo-Fc* map is contoured at 1.5σ (green). (G) Superposition of finger-tips region of apo (light blue) and YM155 bound (pink) structures. The zinc ion is shown as a (blue or pink) sphere

To elucidate the inhibitory mechanism of YM155, we solved the crystal structure of SARS-CoV-2 PLpro^C111S^-YM155 complex to a resolution of 2.1 Å by soaking YM155 into the SARS-CoV-2 PLpro^C111S^ crystals. The structure reveals two common YM155 binding sites on each molecule, including the substrate-binding pocket and the ISG15 binding site (Fig. 5A, 5B and 5E). The interaction between YM155 and PLpro is stabilized through a network of interactions, including hydrophobic interactions, π-stacking interactions and hydrogen bonds.

At the substrate-binding pocket, YM155 forms hydrophobic interactions with the side chains of P248 and with the aromatic rings of Y264 and Y273 (Fig. 5D). These residues are located in the pocket and ordinarily accommodate the leucine residue at P4 position of PLpro substrates (Fig. S6C) (Klemm et al., 2020). The YM155 binding does not induce any significant conformational movement in these residues. The most dramatic change is observed at residue Y268 on the BL2 loop. Upon YM155 binding, the main chain atom of Y268 forms a hydrogen bond with the inhibitor. In the apo structure, the aromatic ring of Y268 swings away from the pocket. In the YM155-bound structure, the aromatic ring of Y268 inserts into the pocket and forms a π-stacking interaction with YM155, thus clamping the inhibitor to the protease (Fig. 5C and 5D). Together with Y268, YM155 occupies the P4 position of PLpro substrate and blocks substrate entry (Fig. S6C). Movements of the homologous BL2 loop in the deubiquitinating enzymes and CoV PLpros upon substrate or inhibitor binding have been observed previously(Hu et al., 2005; Ratia et al., 2008; Lei and Hilgenfeld, 2016). In SARS-CoV PLpro, GRL 0617-binding also induces closure of this loop which helps the inhibitor interact with the protease (Ratia et al., 2008).

In addition, another YM155 binding site was observed near the thumb domain (Fig. 5E). This YM155 molecule mainly interacts with F69 and H73 of SARS-CoV-2 PLpro through hydrophobic interactions. Interestingly, in the ISG15-bound PLpro structure, PLpro F69 is a critical residue involved in hydrophobic interactions to ISG15 (Fig. S6A and 5B) (Shin et al., 2020).

Unexpectedly, another extra YM155 binding site was observed at the zinc-finger motif of chain B with unambiguous electron density (Fig. 5F). The acetic acid methyl ester group of the inhibitor inserts into the cleft, forming a hydrogen bond with Q195. A second hydrogen bond is formed between YM155 naphthoquinone group and T225, which induces T225 Cɑ to move into the cleft by ~3.6 Å (Fig. 5G). This movement leads to positional changes of C224 and C226 and a conformational change in the zinc finger region compared with the apo structure (Fig. 5G). The inhibitor is further stabilized by hydrophobic interactions with the side chains of C192 and C226.

Comparison of the unbound and YM155-bound structures showed that the overall structure is similar between these two structures (r.m.s.d 0.2 Å between the A polypeptides and 0.4 Å between B polypeptides) with two local conformational changes, both induced by inhibitor binding.

## Discussion

Repurposing already approved drugs with favourable properties including bioactivity, biosafety and bioavailability is an excellent strategy for finding new indications. This is because it is highly efficient due to the low-costs required for deployment (i.e., such drugs have already passed all phases of clinical trials) (Pushpakom et al., 2019). High-throughput screening targeting SARS-CoV-2 PLpro by a fluorescence-based enzymatic assay is a new opportunity for COVID-19 drug discovery. Here, we discovered four drug leads that inhibit SARS-CoV-2 PLpro and viral replication.

YM155 is a novel imidazolium-based inhibitor of the antiapoptotic protein survivin (low levels in normal tissue, whereas highly expressed in cancer) and has been investigated for potential treatment of various types of tumors including renal cell carcinoma, lung cancer and prostate cancer (Nakahara et al., 2007; Iwasa et al., 2008; Guo et al., 2015). According to Phase 1 and Phase 2 clinical trials, YM155 is well tolerated in humans and has no serious side effects (Satoh et al., 2009; Kelly et al., 2013). The crystal structures revealed that YM155 has three binding sites on SARS-CoV-2 PLpro. The first one is located at the entrance of the substrate binding pocket and blocks substrate entry to the active site. The second site is located at the thumb domain of PLpro and happens to be the interface between PLpro and ISG15 in the ISG15-bound PLpro structure (Fig. S6B) (Shin et al., 2020). ISG15 is a ubiquitin like protein that significantly upregulated in antiviral response. It can be conjugated to signaling proteins (e.g., IRF3) and therefore enhance antiviral signaling pathways (Shi et al., 2010). Upon SARS-CoV-2 infection, PLpro acts as a protease to cleave ISG15 from IF3 and attenuates type I interferon responses (Shin et al., 2020). Once YM155 partially occupies the interface between PLpro and ISG15, it is likely to decrease the binding between PLpro and ISG15 and therefore enhance the innate immune response.

The third YM155 binding site is located at the zinc finger motif, an essential site for the proteolytic and deubiquitinating activity of PLpro (Herold et al., 1999; Klemm et al., 2020). It has been previously reported that mutating any of four Zn^2+^-coordinating Cystine residues would inactivate the proteolytic activity of PLpro, implying that it might be involved in substrate-binding (Herold et al., 1999). Indeed, it has been shown in the ubiquitin-bound PLpro structure that the zinc-binding fingers subdomain supports ubiquitin binding (Fig. S7A) (Klemm et al., 2020). YM155 causes conformational change of the zinc finger by interacting with both the side chain of T225 through a hydrogen bond and the cysteine residues through hydrophobic interactions. This might perturb the stability of the zinc finger motif and consequently affect enzyme activity. It is worth noting that this YM155 molecule is located at the interface between B chain and the neighboring A chain from another asymmetric unit (Fig. S7B). Thus, we cannot exclude the possibility that binding at this site might be induced by crystal packing. Further biochemical study is required to determine whether this YM155 binding site inhibits enzyme activity.

This unique binding mode demonstrates that how YM155 can recognize three “hot spots” on the drug target, which synergizes to impose potent inhibition. Our structural data also provides a new lead for drug development against SARS-CoV-2 PLpro.

Natural products are also excellent sources for the discovery of pharmacologically active small molecules. *Salvia miltiorrhiza* is an herb that has been widely used in traditional Chinese medicine and has been approved by China FDA (CFDA) for treating cardiovascular diseases (Ren et al., 2019). More than 15 phenolic acids and 30 diterpene compounds have been isolated from *Salvia miltiorrhiza*, including cryptotanshinone and tanshinone I (Zhou et al., 2005). Pharmacological studies indicate that tanshinones have antioxidative, neuroprotective, anti-tumor and anti-inflammatory activities (Jiang et al., 2019). As a patented Chinese medicine, “Xuebijing” (with *Salvia miltiorrhiza* as its main component) has been included in the Chinese clinical guidelines for COVID-19 pneumonia treatment and has shown good clinical efficacy (Tong et al., 2020; Zhang et al., 2020). Cryptotanshinone and tanshinone I are structurally similar molecules and may possess similar effects on the inhibition of SARS-CoV-2 PLpro. Our data implies tanshinones or structurally similar derivatives could be used in the treatment of COVID-19.

As evidenced by successful treatment regimens for HIV or HCV, combination therapies can provide an effective defense against infectious diseases (Gelman and Glenn, 2011; Lu et al., 2018). Hence, we speculate that utilizing a cocktail of multiple drugs aiming at different targets (such as PLpro, M^pro^ and RdRp) is a reasonable strategy for treating CoV-associated diseases. From this perspective, the lead compounds we have identified here serve as promising candidates for use in combination with other antivirals against SARS-CoV-2.

## Materials and methods

### Cloning, protein expression and purification of SARS-CoV-2 PLpro WT and C111S mutant

The full-length gene encoding SARS-CoV-2 PLpro (residues 1,564–1,878 of the SARS-CoV-2 polyprotein, GenBank accession number NC_045512) was synthesized (GeneScript) with codon optimization for *Escherichia coli* expression. Untagged PLpro was cloned into the pET-11a vector using ClonExpress II cloning kit (Vazyme). The expression plasmid transformed the *Escherichia coli* Rosetta (DE3) cells which were then cultured in Luria Broth medium containing 100 μg/mL ampicillin at 37 °C. When the cells were grown to OD_600_ of 0.6–0.8, 0.2 mmol/L IPTG and 0.5 mmol/L zinc acetate (Zn(CH_3_COO)_2_) were added to the cell culture to induce protein expression at 16 °C. After 16 h, the cells were harvested by centrifugation at 5,000 rpm. The cell pellets were resuspended in lysis buffer (20 mmol/L Tris-HCl, pH 7.5, 10 mmol/L β-mercaptoethanol), lysed by high-pressure homogenization, and then centrifuged at 50,000 ×*g* for 30 min. The supernatant was subjected to 40% ammonium sulfate fractionation. The suspension was centrifuged at 50,000 ×*g* for 30 min, and the resulting pellet was resuspended in 20 mmol/L Tris-HCl, pH 7.5, 10 mmol/L β-ME, 1 mol/L ammonium sulfate. The dissolved pellet was loaded onto a Phenyl HS 6FF column (Smart-Lifesciences). Fractions containing SARS-CoV-2 PLpro were pooled and further purified by ion exchange chromatography and size exclusion chromatography (Superdex 200 Increase 10/300 GL, GE Healthcare). The elution volume indicates SARS-CoV-2 PLpro is a monomer in solution.

Using the PLpro plasmid as the template, site-directed mutagenesis (C111S) was performed by an overlapping PCR certified strategy with synthetic primers. The SARS-CoV-2 PLpro^C111S^ was expressed and purified using the same protocol as the wild type enzyme.

### High-throughput drug screening and IC_50_ measurement

The activity of SARS-CoV-2 PLpro was measured by a continuous kinetic assay, with the substrate Arg-Leu-Arg-Gly-Gly-AMC (GL Biochem, Shanghai), using wavelengths of 340 nm and 460 nm for excitation and emission, respectively. All assays were performed in 50 mmol/L HEPES, pH 7.5, 2 mmol/L DTT. The assay was started immediately by mixing 0.2 μmol/L SARS-CoV-2 PLpro with different concentrations of substrate (20–100 μmol/L). Fluorescence intensity was monitored with an EnVision multimode plate reader (Perkin Elmer, USA). Initial rates were obtained by fitting the linear portion of the curves to a straight line. When the different compounds were added into the enzymatic reaction mixture, the change of initial rates was calculated to evaluate their inhibitory effect. Four drug libraries, Approved Drug Library (TargetMol), Clinic Compound Library (TargetMol), FDA-approved Drug Library (Selleck) and Natural Product Library (Selleck), which includes over 6,000 compounds, were used. The preliminary screening reaction mixture included 0.2 μmol/L enzyme, 20 μmol/L substrate and 50 μmol/L compound. The compounds of interest were defined as those with a percentage of inhibition over 60% compared with the reaction in the absence of inhibitor. Then, the multi-point serial dilution concentration inhibition assays were performed to further verify the inhibition ability, and four compounds with strong inhibition were selected for IC_50_ determination. IC_50_ values of the drug leads were measured using 0.2 μmol/L enzyme, 20 μmol/L substrate and serial-diluted inhibitors. In order to exclude inhibitors possibly acting as aggregators, detergent-based control was performed by adding 0.01% freshly made up Triton X-100 to the reaction at the same time (Feng and Shoichet, 2006). All experimental data were analyzed using GraphPad Prism. All experiments were performed in triplicate.

### Antiviral assays and cytotoxicity for compounds from high-throughput screening

A clinical isolate of SARS-CoV-2 (nCoV-2019BetaCoV/Wuhan/WIV04/2019) was propagated in Vero E6 cells, and the viral titer was determined as described previously (Zhou et al., 2020b). For qRT-PCR, pre-seeded Vero E6 cells (5 × 10^4^ cells per well) were pre-treated with different concentrations of drugs for 1 h and the virus was subsequently added (MOI of 0.01) to allow infection for 1 h. Next, the virus-drug mixture was removed and the cells were further cultured with fresh drug-containing medium. At 24 h post infection, a volume of 80 μL cell supernatant was collected from each well for viral RNA (vRNA) copies detection by qRT-PCR analysis, and another 80 μL cell supernatant was collected for plaque-reduction assay.

For the plaque-reduction assay, 1 × 10^5^ Vero E6 cells were seeded in a 24-well plate overnight. Upon infection, cell supernatants collected from the above inhibition assay were diluted by a factor of 1000. Then 200 μL diluents were inoculated onto monolayer Vero E6 cells for 1 h. After removing the supernatant, the plate was washed twice with DMEM medium, and cells were incubated with 0.9% agarose. The overlay was discarded at 4 days post infection and cells were fixed for 30 mins in 4% polyoxymethylene and stained with crystal violet working solution. The plaque forming units were then determined.

For immunofluorescence microscopy, the infected Vero E6 cells were fixed at 24 h post infection, and intracellular NP level was probed with rabbit sera against the NP of a bat SARS-related CoV2 as the primary antibody and Alexa 488-labeled goat anti-rabbit IgG (1:500; Abcam) as the secondary antibody, respectively. The nuclei were stained with DAPI.

For cytotoxicity assays, Vero E6 cells were suspended in growth medium in 96-well plates. The next day, appropriate concentrations of drugs were added to the medium. After 24 h, the relative numbers of surviving cells were measured using a Cell Counting Kit-8 (CCK-8, Beyotime) assay in accord with the manufacturer’s instructions.

All experiments were performed in three biological replicates. All the infection experiments were performed at biosafety level-3 (BSL-3). Vero E6 cells were obtained from ATCC (American Type Culture Collection) with an authentication service. Authentication was performed by a morphology check under a microscope and growth curve analysis. All cell lines were tested negative for mycoplasma contamination. No commonly misidentified cell lines were used.

### Crystallization of SARS-CoV-2 PLpro

Purified SARS-CoV-2 PLpro was concentrated to 3–8 mg/mL in 20 mmol/L HEPES, pH 7.5, 100 mmol/L NaCl, 1 mmol/L TCEP for crystallization screening. Crystallization was performed at 18 °C using the sitting-drop vapor-diffusion method. The needle-like crystals of SARS-CoV-2 PLpro were obtained but diffracted weakly (8 Å). Of all the attempts to crystallize the wild type and mutant SARS-CoV-2 PLpro proteins, the SARS-CoV-2 PLpro^C111S^ (4–315) construct gave the best crystals. Additionally, the proteins were supplemented with the 10% additive (No. 26 of Silver Bullets™ from Hampton Research). Optimized PLpro^C111S^ crystals appeared within one day using 0.1 mol/L Tris pH 8.0, 2.0 mol/L Ammonium sulfate as reservoir. Drops were formed by mixing 1 μL of protein and 1 μL of reservoir which were equilibrated against 80 μL of reservoir solution. The cryo-protectant solution contained 80% reservoir liquor and 20% ethylene glycol. The PLpro^C111S^-YM155 complex was obtained by soaking PLpro^C111S^ crystals with YM155 powder for 30 minutes and cryoprotected in reservoir liquor supplemented with 20% glycol and 1 mmol/L ligand.

### X-Ray data collection and structure refinement

SARS-CoV-2 PLpro^C111S^ crystals were flash-frozen in liquid nitrogen and then transferred into a dry nitrogen stream at 100 K for X-ray data collection. The X-ray data were collected on beamline BL17U1 at Shanghai Synchrotron Radiation Facility (SSRF) at 100 K and at a wavelength of 0.9792 Å using an Eiger X 16M image plate detector. Data were processed and scaled by using the program XDS (Kabsch, 2010). Experimental phase information was determined by the single anomalous dispersion (SAD) method using native Zinc anomalous signal. Phases and experimental electron density maps were calculated with the Phenix AutoSol (Terwilliger et al., 2009) within Phenix 1.18.2 (Liebschner et al., 2019). Iterative model building and refinement was completed with Coot 0.8.9 (Emsley et al., 2010) and Phenix REFINE (Afonine et al., 2012). The final *R*_*work*_ and *R*_*free*_ values were 18.47% and 21.34%. All of the residues are visible in electron density maps. The two monomers in the asymmetric unit superimpose with an rms deviation of 0.6 Å for all Cα atoms. The major difference between two monomers is the angle between the N-terminal Ubl domain and the catalytic which is mostly induced by crystal packing. The thumb and palm domains where the active site is located are almost identical.

PLpro^C111S^-YM155 diffraction data were collected on beamline X06SA at Swiss Light Source (SLS) at 100 K and at a wavelength of 1.0000 Å using an Eiger X 16M image plate detector. The PLpro^C111S^-YM155 structure was solved by molecular replacement (MR) with the PHASER (McCoy et al., 2007) within Phenix 1.18.2 (Liebschner et al., 2019) using the apo structure as a search template. The model from MR was subsequently subjected to iterative cycles of manual model adjustment with Coot 0.8 (Emsley et al., 2010) and refinement was completed with Phenix REFINE (Afonine et al., 2012). The final *R*_*work*_ and *R*_*free*_ values were 18.66% and 21.86%. All of the residues are visible in electron density maps. The structure reveals electron density for five YM155 molecules in one asymmetric unit. There are two consistent YM155 binding sites on each monomer: one at the substrate-binding pocket and one at the thumb domain. Another extra YM155 binding site was observed on the zinc-finger motif of molecule B. It is located at the interface between B chain and the neighboring A chain from another asymmetric unit, implying that this binding site might be induced by crystal packing.

## Supplementary Information

Below is the link to the electronic supplementary material.

Supplementary file 1 (PDF 1448 KB)
