# Supplementary material for: High-throughput screening identifies established drugs as SARS-CoV-2 PLpro inhibitors
Source: Protein Cell. 2021 Apr 17;12(11):877–88. doi: 10.1007/s13238-021-00836-9 (PMC8052528; doi:10.1007/s13238-021-00836-9)
Supplement: 13238_2021_836_MOESM1_ESM [file 13238_2021_836_moesm1_esm.pdf]

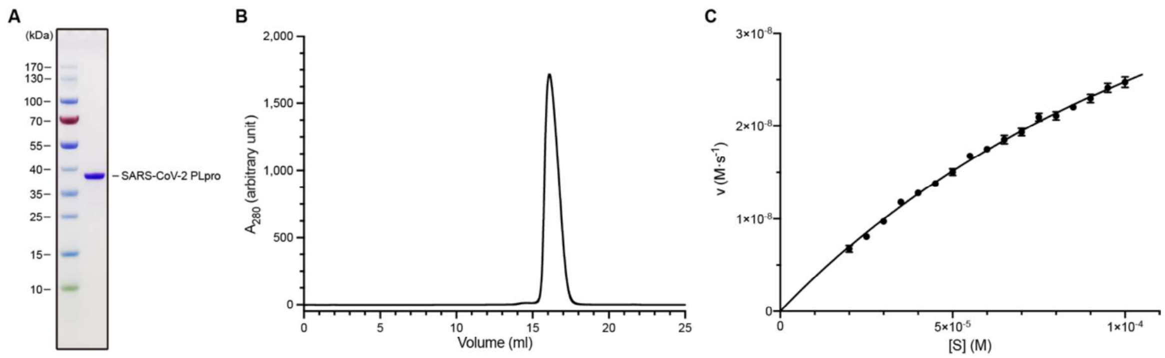

Figure S1. The purification and enzymatic activity of SARS-CoV-2 PLpro. (A) The SDS-PAGE gel of SARS-CoV-2 PLpro. (B) Size-exclusion chromatography profile of SARS-CoV-2 PLpro. (C) Michaelis-Menten Plot for SARS-CoV-2 PLpro. Data is shown as mean ± s.e.m., n = 3 biological replicates.

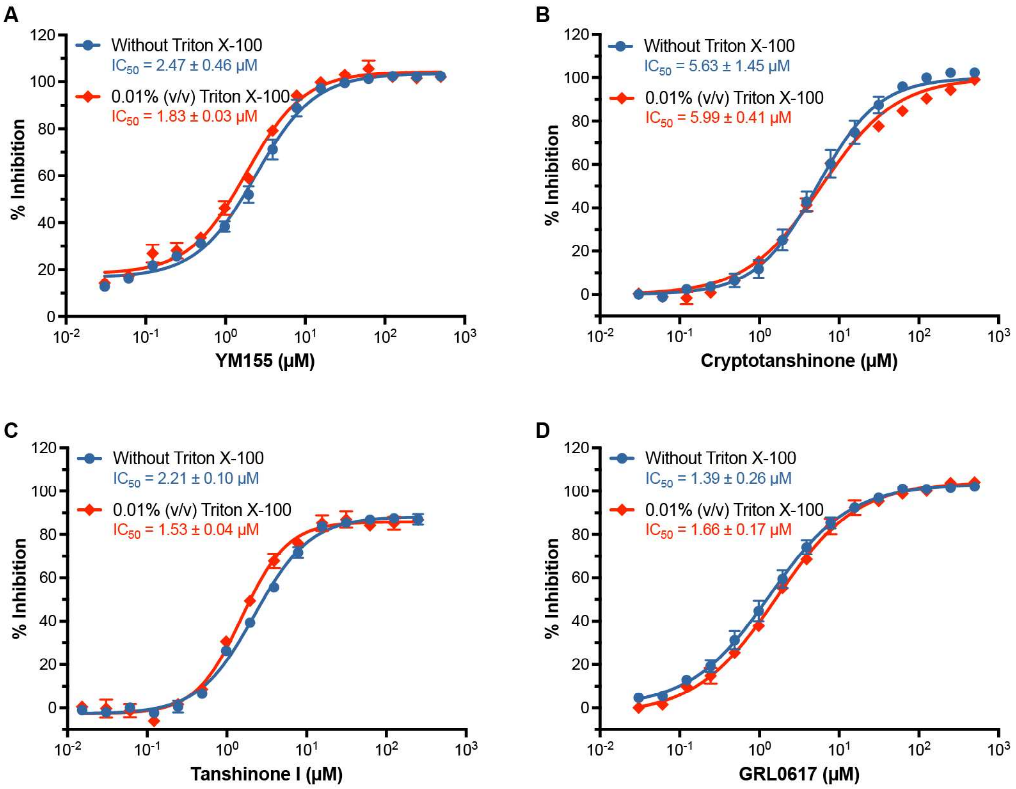

Figure S2. The detergent-based assay for drug leads. (A-D) The IC_{50} values determined by in the presence (red) or absence (blue) of 0.01% (v/v) Triton X-100, which showed that adding detergent did not affect the effects of inhibitors. All data are shown as mean ± s.e.m., n = 3 biological replicates.

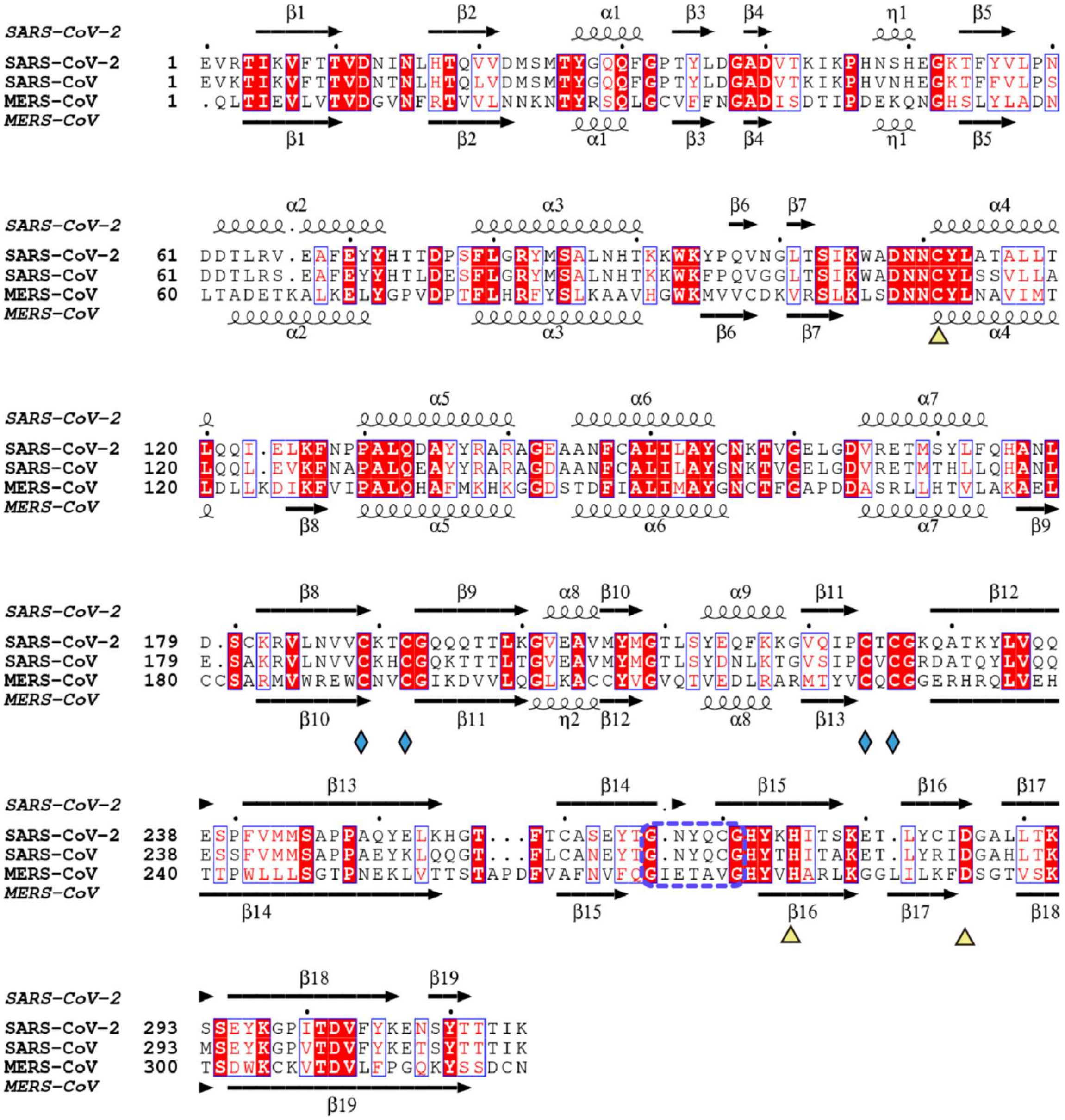

Figure S3. Sequence alignment of PLpros from SARS-CoV-2, SARS-CoV and MERS-CoV. Secondary structure elements of SARS-CoV-2 PLpro (top) and MERS-CoV PLpro（bottom: PDB ID 4WUR）are indicated. SARS-CoV-2 PLpro shares sequence identity of 82% and 29% with SARS-CoV PLpro and MERS-CoV PLpro, respectively. The conserved cysteines involved in zinc binding are marked by the cyan rhomboids. The catalytic triad is marked by yellow triangles. The BL2 region is marked by a blue dashed rectangle.

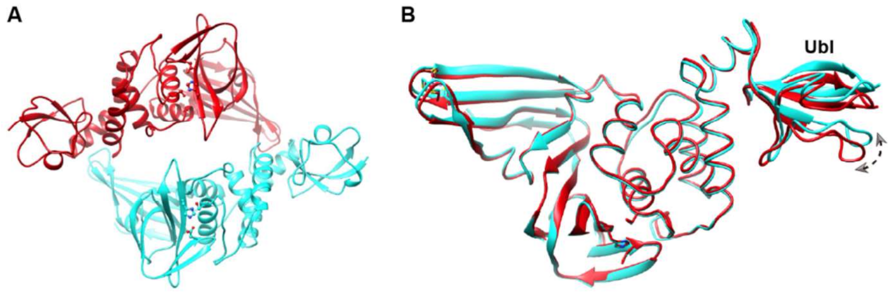

Figure S4. Crystal structure of SARS-CoV-2 PLproC111S. (A) The asymmetric unit structure of SARS-CoV-2 PLpro. (B) Superimposition of the two protomers. The region with significant deviation is marked by a dashed arrow.

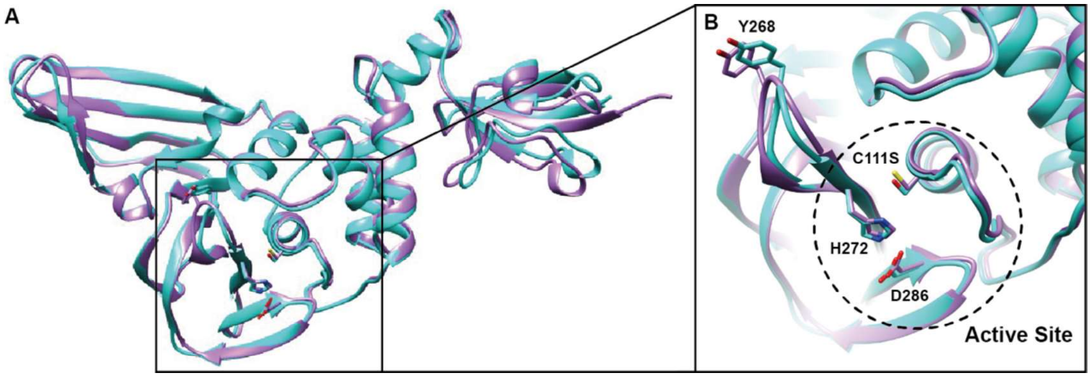

Figure S5: Comparison of SARS-CoV-2 PLpro^{C111S} with native SARS-CoV-2 PLpro structure. (A) Superposition of SARS-CoV-2 PLpro^{C111S} (cyan) with native SARS-CoV-2 PLpro(purple) (PDB ID 6WZU). (B) Magnified view of the active sites.

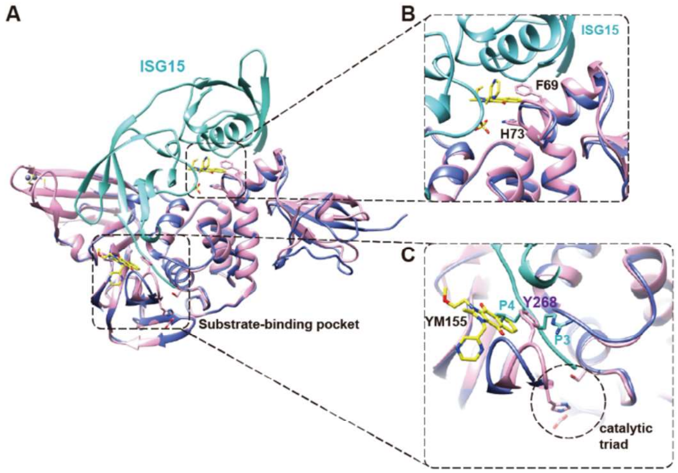

Figure S6. Comparison of SARS-CoV-2 PLpro^{C111S}-YM155 with SARS-CoV-2 PLpro^{C111S}-ISG15 structure. (A) Superposition of YM155(yellow) bound SARS-CoV2 PLpro (pink) with ISG15(cyan) bound SARS-CoV-2 PLpro(blue) (PDB ID 6YVA). (B) Magnified view of the substrate-binding pockets. (C) Magnified view of the substrate-binding pockets showing that YM155 and Try268 of PLpro occupy the P4 position of the substrate.

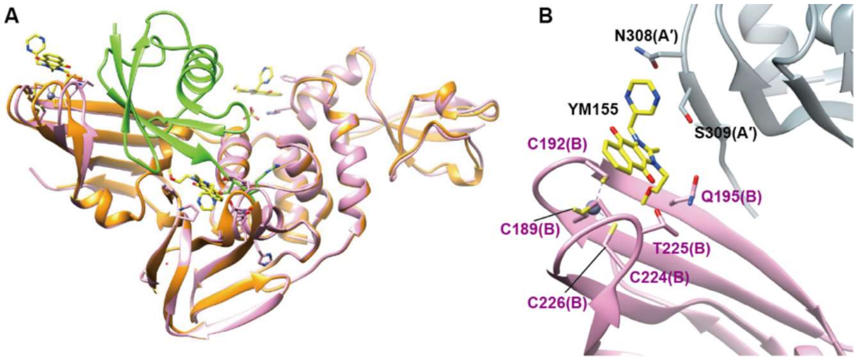

Figure S7. YM155 binding site at the Zinc-binding motif. (A) Superposition of YM155(yellow) bound SARS-CoV2 PLpro (pink) with ubiquitin(green) bound SARS-CoV-2 PLpro(orange) (PDBID: 6XAA). (B) The M155 molecule is located at the interface between chain B chain the neighboring chain A from another asymmetric unit (grey).

Extended Data Table 1 Data collection and refinement statistics

	$PLpro^{C111S}$	$PLpro^{C111S}$-YM155
	PDB code: 7D7K	PDB code: 7D7L
**Data Collection**		
Space group	$P6_522$	$P6_522$
Cell dimensions		
a, b, c (Å)	115.00, 115.00, 254.19	117.56, 117.56, 257.30
α, β, γ (°)	90, 90, 120	90, 90, 120
Resolution (Å)	49.28 - 1.90 (1.97 - 1.90)	48.49 - 2.11 (2.19 - 2.11)
R_{merge}	0.1286 (3.60)	0.2783 (4.26)
R_{meas}	0.1303 (3.65)	0.2819 (4.32)
R_{pim}	0.021 (0.57)	0.046 (0.66)
$I / \sigma I$	27.20 (1.68)	18.75 (1.58)
$CC1/2$	1 (0.712)	0.997 (0.665)
Completeness (%)	98.73 (98.13)	99.95 (99.98)
Redundancy	39.5 (40.6)	39.6 (41.0)
**Refinement**		
Resolution (Å)	49.28 - 1.90	48.49 - 2.11
No. reflections	79,119 (7,732)	61,146 (5,981)
R_{work} / R_{free}	0.1847 / 0.2134	0.1866 / 0.2186
No. atoms		
Protein	4929	4910
Ligand/ion	107	222
Water	589	396
B-factors		
Protein	47.64	50.84
Ligand/ion	61.79	80.35
Water	53.65	51.68
R.m.s. deviations		
Bond lengths (Å)	0.019	0.017
Bond angles (°)	1.50	1.38
Methods		
Favored (%)	97.10	97.74
Allowed (%)	2.90	2.26
Outliers (%)	0	0
